# Perturbation of the MetJ regulon impacts the consequences of 2-aminoacrylate stress in Salmonella enterica

**DOI:** 10.1099/mic.0.001572

**Published:** 2025-06-24

**Authors:** Bryce R. Sawyer, Wangchen Shen, Diana M. Downs

**Affiliations:** 1 Department of Microbiology, University of Georgia, Athens, GA 30602-2605, USA

**Keywords:** 2-aminoacrylate stress, MetJ, MetR, RidA

## Abstract

In the absence of the broadly conserved deaminase RidA (Reactive intermediate deaminase A), *Salmonella enterica* and other organisms accumulate the reactive enamine species 2-aminoacrylate (2AA). Free 2AA, generated from serine by the serine/threonine dehydratase IlvA, reacts with and covalently inactivates a subset of pyridoxal 5′-phosphate-dependent enzymes. The metabolic stress caused by 2AA generates growth defects in *S. enterica*, including (i) when l-alanine is used as a nitrogen source, (ii) when pyruvate is used as a carbon source or (iii) in the presence of exogenous serine. Although the enzymatic targets of 2AA are consistent between growth conditions, the consequences of 2AA-dependent damage differ depending on the distribution of metabolic flux required in different conditions. Analysing the suppressors of a *ridA* mutant has furthered our understanding of the RidA stress paradigm and, more generally, how a metabolic network responds to perturbation. Many such suppressors modulate the metabolic network to eliminate 2AA production by IlvA. Here, we describe that eliminating the MetJ transcriptional repressor allows a *ridA* mutant to grow in the presence of 2AA stress in each of the three conditions. The mechanisms by which a Δ*metJ* suppresses a *ridA* mutant are nuanced and medium-dependent, emphasizing that consequences of 2AA stress differ based on environmental and metabolic context.

## Importance

RidA (Reactive intermediate deaminase A) is an enamine deaminase that has been characterized for preventing 2-aminoacrylate (2AA) stress in *Salmonella* and other micro-organisms. 2AA causes damage to many pyridoxal 5′-phosphate (PLP)-dependent enzymes and consequently perturbs the metabolic network of the organism. Here, we show that eliminating the transcription factor MetJ suppresses the growth defect of a *ridA* mutant in each of the three conditions, by different mechanisms. This work addresses the ripple effects caused by perturbation of the metabolic network when enzymes are damaged by 2AA and, as such, contributes to understanding the components and connections in microbial metabolism.

## Data Availability

All relevant data are included in the content of this manuscript.

## Introduction

Bacterial metabolism consists of a complex network of biochemical reactions that function in unison to generate the robust physiology that is the hallmark of these organisms. The resistance of the metabolic network to perturbations, which can be caused by exogenous or endogenous stresses, allows micro-organisms to thrive in changing environments. Resistance to perturbations can be facilitated by inherent robustness and redundancy, mutations or environmental cues that modify flux distribution. In many cases, the system can retain function despite a disruption in the normal equilibrium of the components. Biochemical and genetic analyses of mutants that are sensitive to a particular stressor can provide insights into the roles of individual components of the metabolic network and their integration. Analysing the response of cells to such a stressor can help define the architecture of the metabolic network and how it can be remodelled to maintain fitness under changing conditions.

**Fig. 1.**
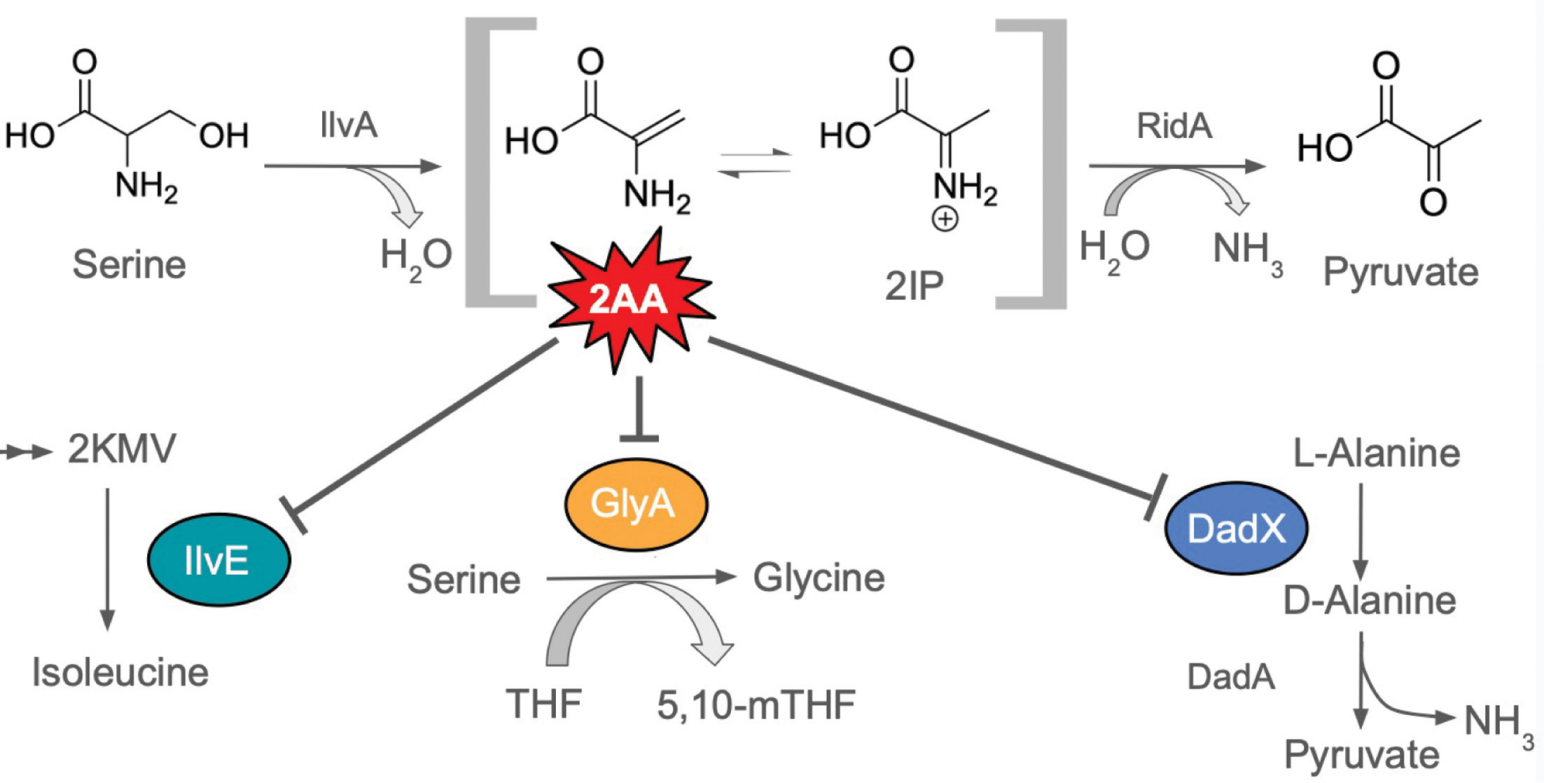
The RidA paradigm and relevant targets of 2AA stress in *S. enterica*. The PLP-dependent serine/threonine dehydratase IlvA (EC 4.3.1.19) generates 2AA, which tautomerizes to 2-iminopropionate (2IP). 2AA can be deaminated by water, or the RidA deaminase, to form pyruvate. In the absence of RidA, 2AA persists in the cell long enough to damage PLP-dependent enzymes. Depending on the unique structure of an organism’s metabolic network and the nutrient composition of its surroundings, damage to certain key enzymes may generate a growth defect. In *S. enterica*, relevant targets of 2AA include, but are not limited to, (**i**) IlvE (EC 2.6.1.27), an aminotransferase required for the last step of isoleucine biosynthesis; (ii) GlyA (serine hydroxymethyl transferase, EC 2.1.2.1), the predominant source of glycine and one-carbon units; and (iii) DadX (EC 5.1.1.1), the catabolic alanine racemase required for l-alanine utilization. Damage to these enzymes has been associated with growth defects on minimal media with pyruvate provided as the sole carbon source, exogenous serine or l-alanine provided as the sole nitrogen source, respectively. Abbreviations: 2KMV, 2-keto-3-methylvalerate; THF, tetrahydrofolate; 5,10-mTHF, 5,10-methylene-tetrahydrofolate; DadA, d-amino acid dehydrogenase (EC 1.4.5.1).

RidA (Reactive intermediate deaminase A) is the founding member of the highly conserved Rid (YjgF/YER057c/UK114) protein family [1,2]. Members of the RidA subfamily are found across all domains of life and catalyse the deamination of enamine/imine metabolites, specifically those that can be deaminated by solvent water. In the absence of RidA, *Salmonella enterica* and other organisms accumulate the reactive metabolite 2-aminoacrylate (2AA) [3,5]. The 2AA stress paradigm provides a model to characterize how a metabolic network can absorb various stresses and the strategies embedded in the design of the system that retain the fitness of the organism (Fig. 1). 2AA is an obligate intermediate in the reaction catalysed by some pyridoxal 5′-phosphate (PLP)-dependent enzymes, notably the serine/threonine dehydratase IlvA (EC 4.3.1.19). When IlvA uses serine instead of its preferred threonine substrate, 2AA is generated [3]. Deamination of 2AA occurs spontaneously in solvent water, but *in vivo* the absence of RidA results in the accumulation of this reactive enamine. In this situation, free 2AA molecules inactivate a broad assortment of sensitive PLP-dependent enzymes with an irreversible covalent modification [4,6,8]. The level of enzyme damage can be such that it results in a growth defect specific to the role of the damaged enzyme [3,8,10].

Three conditional growth defects of a *ridA* mutant of *S. enterica* are the lack of growth when (i) l-alanine is the sole nitrogen source in the absence of the anabolic alanine racemase Alr, (ii) pyruvate is the sole carbon source and (iii) an exogenous generator of 2AA (i.e. serine) is provided in the medium. These growth defects are caused by 2AA-dependent damage to IlvE (BCAA transaminase, EC 2.6.1.27), GlyA (serine hydroxymethyl transferase, EC 2.1.2.1), DadX (catabolic alanine racemase, EC 5.1.1.1) and/or IscS (cysteine desulfurase, EC 2.8.1.7) [8,10]. Further, in each of these conditions, the relevant 2AA is produced by IlvA acting on serine that was either endogenously generated or exogenously added. Growth in all conditions is restored by addition of isoleucine, which allosterically inhibits IlvA and thus prevents 2AA formation [5,11, 12]. Suppressor analyses have been used successfully to identify connections in the metabolic network and the effect of 2AA-dependent perturbations on flux through the metabolic system. Several mutations that suppress *ridA* mutant phenotypes act by altering flux in a way that lowers the amount of 2AA synthesized [13,16]. Prior to this work, a single suppressor had been identified that restored the growth of a *ridA* mutant in the presence of serine but failed to decrease the level of 2AA stress that the cell was experiencing. This suppressor encoded a variant of IscS with less sensitivity to 2AA attack and was identified in *Pseudomonas aeruginosa* [8]. The study here was initiated to continue the analyses of metabolic changes that allow bypass of 2AA stress and better understand metabolic pathway integration in *S. enterica*. An observation that deleting the methionine repressor *metJ* restored growth to an *alr ridA* double mutant of *S. enterica* when l-alanine was provided as the sole nitrogen source was investigated. The data showed that lack of *metJ* suppressed multiple phenotypes of a *ridA* mutant and did so by at least three related mechanisms, none of which reduced the 2AA level in the cell.

## Methods

### Strains, media and chemicals

Strains used in this study are derivatives of *S. enterica* serovar Typhimurium LT2 (denoted *S. enterica* throughout) and are listed in Table 1. Strains were grown in Nutrient Broth (NB; Difco; 8 g l^−1^, 5 g l^−1^ NaCl) as a rich medium. Minimal medium was no-carbon E (NCE) medium supplemented with 1 mM MgSO_4_, trace minerals and 11 mMd-glucose (or 22 mM pyruvate as indicated) as the sole carbon source [17]. NCE medium provides 16 mM ammonium as the available nitrogen source. No-carbon no-nitrogen medium was supplemented with MgSO_4_, trace minerals and d-glucose (11 mM) as the carbon source and l-alanine (5 mM) as the sole nitrogen source. Other supplements were added as indicated: glycine (1 mM), HMP (100 nM), dl-homocysteine (0.2 mM), l-isoleucine (0.3 mM), l-methionine (0.3 mM), l-serine (5 mM), thiamine (100 nM), THZ (100 nM) and l-tyrosine (0.08 mM). Chemicals were purchased from MilliporeSigma (St. Louis, MO).

**Table 1.** Strain list

Strain	Genotype	Source
DM9404	WT	Laboratory collection
DM3480	*ridA3*::MudJ*	Laboratory collection
DM13760	*dadX121*::Cm	Laboratory collection
DM14178	*alr51*::Cm	Laboratory collection
DM14179	*alr51*::Cm *ridA3*::MudJ	Laboratory collection
DM17594	Δ*alr ridA3*::MudJ	This study
DM17613	*dadX121*::Cm Δ*alr ridA3*::MudJ	This study
DM17632	*alr*::Km	This study
DM17776	*metJ*::Kan (nt4322746-4323098)†	This study
DM17781	Δ*alr ridA1*::Tn*10*d(Tc)‡	This study
DM17788	Δ*alr ridA1*::Tn*10*d(Tc) *metJ*::Kan	This study
DM17972	Δ*alr ridA3*::MudJ *metC*::Tn*10*d(Tc)	This study
DM17973	Δ*alr ridA1*::Tn*10*d(Tc) *metJ*::Kan *dadX121*::Cm	This study
DM17984	Δ*alr ridA*::Cm *metJ*::Kan	This study
DM18006	Δ*alr ridA*::Cm *metJ*::Kan *metC*::Tn*10*d(Tc)	This study
DM18540	*alr*::Km *metR*::Tn10d(Tc)	This study
DM18541	Δ*alr ridA3*::MudJ *metR*::Tn*10*d(Tc)	This study
DM18542	Δ*alr ridA*::Cm *metJ*::Kan *metR*::Tn*10*d(Tc)	This study

*MudJ refers to the MudJ1734 insertion element [39].

†From BEI collection (SGD 156/157 Kan, B11) [40].

‡Tn*10*d(Tc) refers to the transposition-defective mini-Tn*10* (Tn*10*Δ16Δ17) [41].

### Strain construction

Mutations were moved between *S. enterica* strains as needed using transduction. Transductions were carried out with the high-frequency general transducing mutant of bacteriophage P22 (HT105/1, *int*-201) as described previously [18,20]. The transductants were confirmed to be phage-sensitive and have relevant phenotypes prior to storage. Phage λ Red recombineering [21] was adapted for *S. enterica* to generate gene deletions. Deletions were confirmed by colony PCR.

### Growth analyses

Cultures were started from a single colony and grown overnight in NB medium with shaking at 37 °C. Cells were pelleted and resuspended in an equal volume of sterile saline. Cell suspensions (5 µl) were used to inoculate the indicated medium (195 µl), and growth was monitored as the change in OD_650_ over time using a BioTek ELx808 plate reader (BioTek Instruments, Winooski, VT) with a slow shaking speed (210 r.p.m.). Data were plotted using GraphPad Prism version 10.2. Growth experiments were performed with three independent biological replicates unless otherwise stated.

### Branched-chain amino acid aminotransferase (IlvE) assay

Cultures were grown overnight in NB medium with shaking at 37 °C before cells were pelleted and resuspended in an equal volume of sterile saline. Cell suspensions (500 µl) were used to inoculate 20 ml of the relevant medium, and the resulting cultures were grown to late log phase over 8–12 h. Cells were then pelleted, washed in NCE medium and pelleted a second time prior to being frozen at −80 °C until use. Cell pellets were thawed and resuspended in 400 µl of 50 mM potassium phosphate buffer (pH 7.5) and lysed mechanically with a Constant Systems Limited One Shot (UK) at 20 kpsi. Cell lysate was clarified by centrifugation at 17,000 ***g*** for 20 min at 4 °C.

Branched-chain amino acid aminotransferase was assayed as described previously [22]. Briefly, cell-free extract (100 µl) was added to an 80 µl reaction mixture (4 µl of 0.5 M *α*-ketoglutarate; 76 µl of 50 mM potassium phosphate buffer, pH 7.5). The reaction was initiated with the addition of l-isoleucine (to 20 mM) and incubated at 37 °C for 20 min. Resulting 2KMV was derivatized by adding 2,4-dinitrophenylhydrazine (200 µl) to yield a hydrazone, which was treated with 0.5 N HCl. The resulting organic layer was removed and mixed with 1.5 N NaOH to allow the formation of an aqueous layer containing a chromophore with an absorbance at 540 nm. The aqueous layer was extracted, and absorbance was measured using a SpectraMax M2 microplate reader (Molecular Devices). 2KMV concentration was quantified by use of a standard curve. The protein concentration of each lysate was determined using the bicinchoninic acid assay (Pierce). Activity is reported as nanomoles of 2KMV per milligram of protein per cell lysate. Data plotting and statistical analyses were conducted using GraphPad Prism version 10.2.

## Results

### Loss of *metJ* suppresses *ridA* mutant phenotypes

**Fig. 2.**
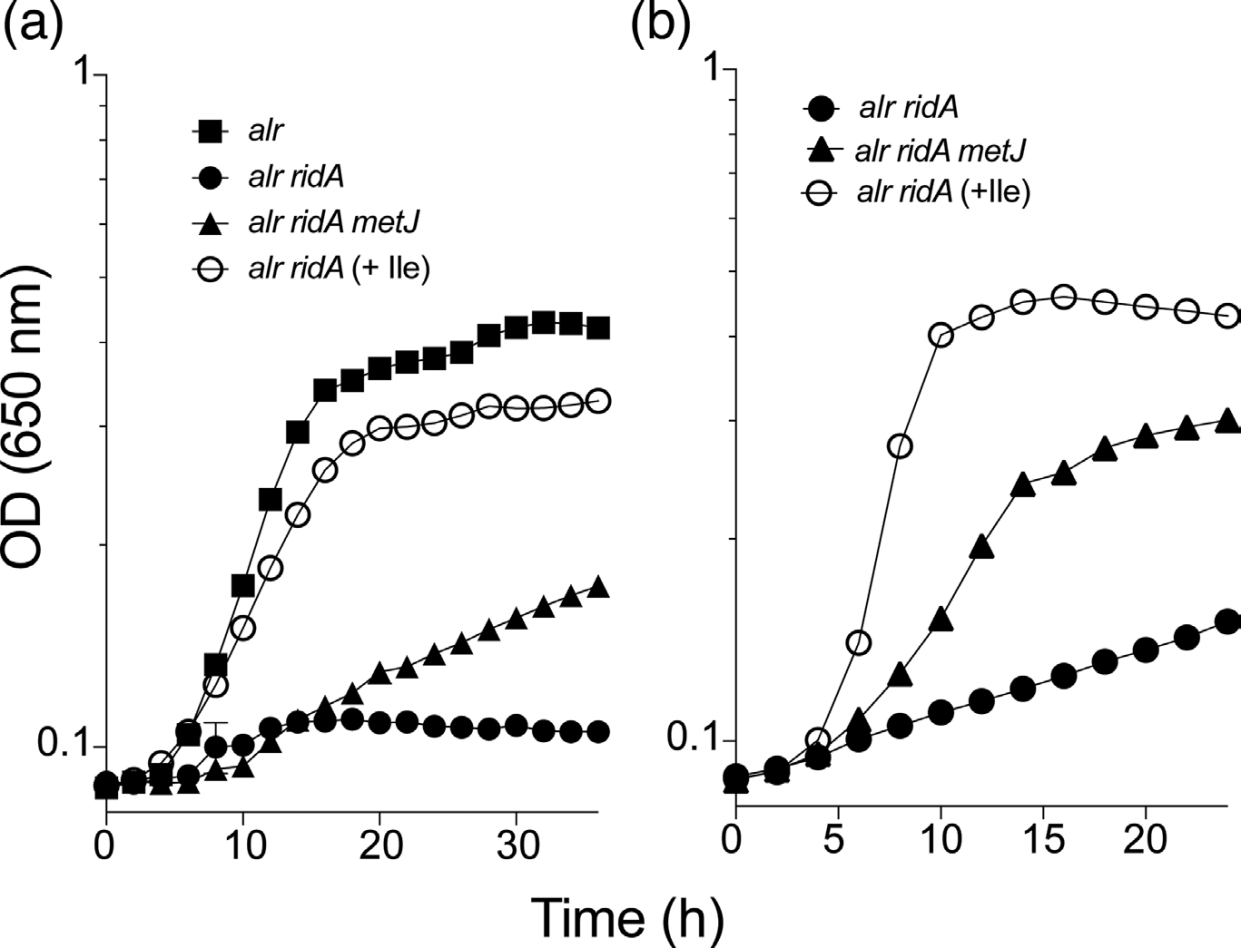
Deletion of *metJ* improves the growth of an *alr ridA* mutant. Strains with the indicated genotypes [*alr* (squares; DM14178), *alr ridA* (circles; DM17781) and *alr ridA metJ* (triangles; DM17788)] were grown on (**a**) minimal glucose medium with l-alanine (5 mM) as the sole nitrogen source and (**b**) minimal pyruvate medium. Control growth is shown as *alr ridA* in the presence of isoleucine in each medium. Growth was monitored by following OD at 650 nm over 25–35 h as indicated. Data shown are averages from three biological replicates with standard error bars.

When assessed on minimal glucose medium with l-alanine as the sole nitrogen source, an *alr ridA* mutant strain is unable to grow because of 2AA-mediated damage of the catabolic alanine racemase, DadX [9]. MetJ is a transcriptional regulator, and in its absence, multiple *met* genes are derepressed [23,24]. In *Escherichia coli*, a *metJ* mutation bypassed the requirement for DadX to utilize l-alanine as the sole nitrogen source by derepressing *metC* [25]. The *E. coli* MetC (cystathionine *β*-lyase) had enough alanine racemase activity to allow catabolism of l-alanine for growth. Based on these data, a *metJ* insertion mutation was transduced into the *S. enterica ridA* mutant backgrounds. In fact, a *metJ* mutation allowed significant growth of the *alr ridA* mutant strain (Fig. 2a). The results in *E. coli* suggested a mechanism for this suppression of the *alr ridA* mutant. However, when a deletion of *dadX* was present in the *alr ridA metJ* mutant, growth with l-alanine as the sole nitrogen source was eliminated – despite the addition of the d-alanine (0.3 mM) required by Δ*dadX* mutant [26] (data not shown). Thus, suppression of the *alr ridA* defect by a *metJ* mutation in *S. enterica* required DadX, suggesting that MetC was not involved. Further, deleting *metC* in the *alr ridA* and *alr ridA metJ* mutants did not impact the significant growth allowed by the *metJ* mutation on medium with l-alanine as the sole nitrogen source and supplemented with methionine (data not shown). Together, these results nullified the hypothesis that growth allowed by a *metJ* mutation was due to a moonlighting activity of MetC in *S. enterica*.

A *ridA* mutant fails to grow with pyruvate as the sole carbon source due to 2AA accumulation [27]. A *metJ* mutation restored significant growth to the *alr ridA* mutant on minimal pyruvate medium (Fig. 2b). The 2AA stress preventing an *alr ridA* mutant from using l-alanine as a nitrogen source, or pyruvate as a carbon source, is due to IlvA acting on the endogenous pool of serine [4,10, 27]. As expected, isoleucine restored full growth to the *alr ridA* mutant strain in both media (Fig. 2), since 2AA generation is prevented by allosteric inhibition of IlvA [11,28].

### A *metJ* mutation does not decrease 2AA stress

In a simple scenario, a *metJ* mutation could suppress effects of a *ridA* mutation by decreasing intracellular 2AA stress, either by reducing IlvA-mediated generation of 2AA or by sequestering existing 2AA. Both mechanisms for suppressing *ridA* phenotypes have been described [13,15, 29]. The short half-life of 2AA prevents it from being measured directly in the cell. Instead, the activity of a characterized target of 2AA, IlvE, is used as a proxy for intracellular 2AA levels. Decreased IlvE activity reliably corresponds with increased 2AA stress [5,11, 28]. IlvE activity was measured in each of three strains grown in minimal glucose medium (Table 2). Consistent with previous studies, the *alr ridA* mutant had lower IlvE activity than the parental *alr* mutant strain (71 vs 147 nmol mg^−1^, respectively). Addition of isoleucine to the growth medium restored IlvE activity (140 nmol mg^−1^) due to allosteric inhibition of IlvA, preventing the formation of 2AA [11,28]. Importantly, IlvE activity in the *alr ridA metJ* mutant was not significantly different from that of the *alr ridA* mutant strain (83 vs 71 nmol mg^−1^, respectively). IlvE activity was also restored in the *metJ* mutant strain by isoleucine (161 nmol mg^−1^). These data indicated that a *metJ* deletion does not decrease 2AA stress and suggested that lack of the transcriptional regulation was altering the metabolic landscape such that it supported growth in the presence of 2AA stress. This result also explained the relatively weak suppression of growth on l-alanine, since DadX remains compromised by 2AA.

**Table 2.** A *metJ* mutation does not reduce 2AA stress in a *ridA* mutant

		BCAA transaminase activity†	
Strain	Genotype*	Minimal	Minimal+Ile
DM14178	WT	147±32	151±9
DM17781	*ridA*	71±33	140±11
DM17788	*ridA metJ*	83±11	161±16

*Each strain carries an *alr* mutation in addition to the lesion(s) listed.

†BCAA transaminase (IlvE) activity is reported as nanomoles of 2-keto-3-methylvalerate (2KMV) per milligram of protein.

Strains were grown in minimal glucose medium with or without l-isoleucine (0.3 mM) to a final OD_650_ of ~0.8. The branched-chain amino acid transaminase activity was measured as described in the text. Data shown are the mean and sd of two biological replicates assayed in three technical replicates. IlvE activity of the *ridA metJ* mutant strain was not significantly different from that of the *ridA* mutant strain, and both were significantly lower than that of the corresponding WT strain (*P*≤0.05 as determined by Sidak’s multiple comparisons test).

### Loss of *metJ* allows growth in 2AA stress by more than one mechanism

Despite differences in the metabolic network of the cells growing on pyruvate/NH3 vs glucose/L-Ala, a *metJ* lesion restored growth to the *ridA* mutant in both media. Loss of *metJ* increases methionine biosynthesis [23,24, 30]. Exogenous methionine had no beneficial effect on the growth of the strain with l-alanine (data not shown), but it improved the growth of an *alr ridA* mutant when pyruvate was the sole carbon source (Table 3a). On minimal pyruvate medium, methionine increased the yield of the *alr ridA* mutant to approximately half the level allowed by isoleucine [OD at 650 nm (OD_650_) of 0.081 to 0.309 and 0.784, respectively]. While a *metJ* mutation increased the final yield on minimal pyruvate (OD_650_ of 0.081 vs 0.441, respectively), addition of methionine provided only a slight additional benefit (OD_650_ of 0.549) to the *metJ*-containing strain. These data supported the conclusion that the *metJ* mutation restored the growth of a *ridA* mutant on pyruvate primarily by increasing methionine biosynthesis.

**Table 3.** Methionine and thiamine suppress a *ridA* mutant

(a)		Cell yield (OD_650_) on medium with:				
Strain	Genotype*	None	Ile	Met	Thi	Met/Thi
DM14178	WT	0.77±0.01	0.79±0.02	0.78±0.01	0.77±0.01	0.74±0.02
DM17781	*ridA*	0.08±0.01	**0.78±0.01**	**0.31±0.01**	**0.30±0.01**	**0.36±0.01**
DM17788	*ridA metJ*	0.44±0.04	**0.56±0.01**	**0.55±0.01**	0.42±0.02	**0.56±0.07**

(b)	Cell yield (OD_650_) on medium with:							
Strain	Genotype*	None	Ile	Thi	HMP	THZ	HMP/THZ	HMP/Tyr
DM14178	WT	0.71±0.02	0.80±0.02	0.77±0.03	0.78±0.01	0.77±0.01	0.70±0.01	0.75±0.01
DM17781	*ridA*	0.05±0.01	**0.80±0.01**	**0.28±0.01**	0.06±0.01	0.08±0.01	**0.26±0.01**	**0.27±0.01**
DM17788	*ridA metJ*	0.43±0.01	**0.60±0.01**	0.49±0.01	0.50±0.01	0.43±0.03	0.44±0.03	0.40±0.01

*All strains carry an *alr* mutation, with the additional lesions (if any) indicated.

Strains were grown on minimal pyruvate media containing supplements as listed: l-isoleucine (0.3 mM), l-methionine (0.3 mM), thiamine (100 nM), HMP (100 nM), THZ (100 nM) and l-tyrosine (80 µM). Final yield was the OD_650_ after ~18 h of continuous growth at 37 °C with shaking. Data shown are the mean and sd of three biological replicates. Data that are bolded significantly differ from the corresponding control grown in an unsupplemented medium. Tables (a) and (b) display data from separate experiments.

The integration of methionine and one-carbon metabolism suggested the hypothesis that a *ridA* mutant growing on pyruvate is limited for S-adenosyl methionine (SAM). Serine hydroxymethyltransferase (GlyA; E.C 2.1.2.1) is the primary generator of 5,10-methylene-tetrahydrofolate and a primary target of 2AA stress in *ridA* mutants [10]. The final step of methionine biosynthesis involves the transfer of a one-carbon unit, suggesting that the synthesis of methionine could be impacted if GlyA is compromised by 2AA. Low methionine synthesis would lead to lowered levels of SAM, which results in compromised thiamine biosynthesis in *S. enterica* [31,32]. Consistently, thiamine restored growth of the *alr ridA* mutant on pyruvate medium to a level like that achieved with methionine (OD_650_ of 0.309 vs 0.296) (Table 3a). The two nutrients were not additive, which is consistent with methionine acting by restoring thiamine synthesis. Thiamine is composed of two independently synthesized moieties, 4-methyl-5-(2-hydroxyethyl)-thiazole (THZ) and 4-amino-5-(hydroxymethyl)-2-methylpyrimidine (HMP). Both the thiazole and pyrimidine moieties were required to restore the growth of the *alr ridA* mutant on minimal pyruvate medium (Table 3b). Significantly, the synthesis of THZ and HMP requires radical SAM enzymes, ThiH (2-imioacetate synthase EC 4.1.99.19) and ThiC (HMP-P synthase, EC 4.1.99.17), respectively. Finally, genetic analysis of *thiH* mutants showed that tyrosine could satisfy a THZ requirement in mutants with reduced SAM binding [31]. Thus, the ability of tyrosine to replace the THZ requirement further supported the conclusion that SAM limitation was the requirement solved with supplementation of methionine or thiamine. In total, these nutritional data supported the conclusion that part of the defect of a *ridA* mutant growing on pyruvate is decreased methionine synthesis, which manifests as low SAM pools and generates a thiamine requirement.

### Loss of *metJ*, but not exogenous methionine, restores growth of a *ridA* mutant with serine

**Fig. 3.**
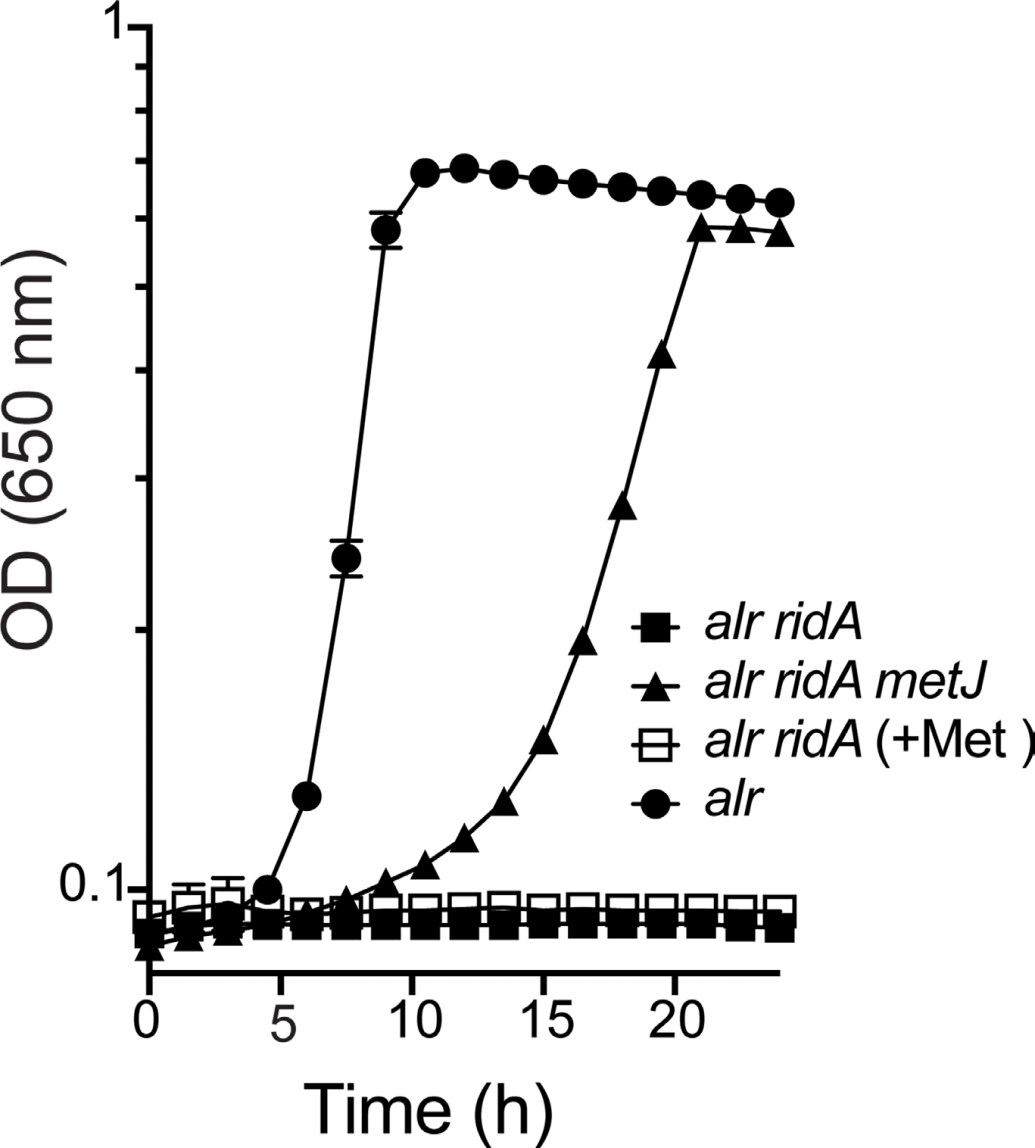
Deletion of *metJ* improves the growth of an *alr ridA* mutant with exogenous serine. Strains with the indicated genotypes [*alr* (circles; DM14178), *alr ridA* (squares; DM17781) and *alr ridA metJ* (triangles; DM17788)] were grown on minimal glucose medium with serine (5 mM). The *alr ridA* mutant was also grown with serine and methionine (open squares). Growth was monitored by OD (650 nm) over 24 h. Data shown are averages from three biological replicates with standard error bars.

Strains lacking *ridA* accumulate 2AA to different levels depending on growth conditions. In conditions above (l-alanine and pyruvate), 2AA is derived from endogenous serine pools acted on by IlvA. Exogenous addition of 2AA generators increases the level of 2AA stress and can thus cause additional phenotypic defects [5,33, 34]. In *S. enterica*, *ridA* mutants fail to grow in the presence of exogenous serine (5 mM) due to the decreased activity of GlyA and IscS caused by 2AA-dependent damage [8,10, 35]. Methionine did not restore growth to a *ridA* mutant in the presence of serine, and yet a *metJ* mutation dramatically increased growth (Fig. 3). These data, in combination with the data from growth on l-alanine, emphasized that loss of *metJ* allows growth in the presence of 2AA stress by a mechanism(s) beyond increasing endogenous methionine pools.

### Homocysteine increases suppression in a MetR-dependent manner

**Fig. 4.**
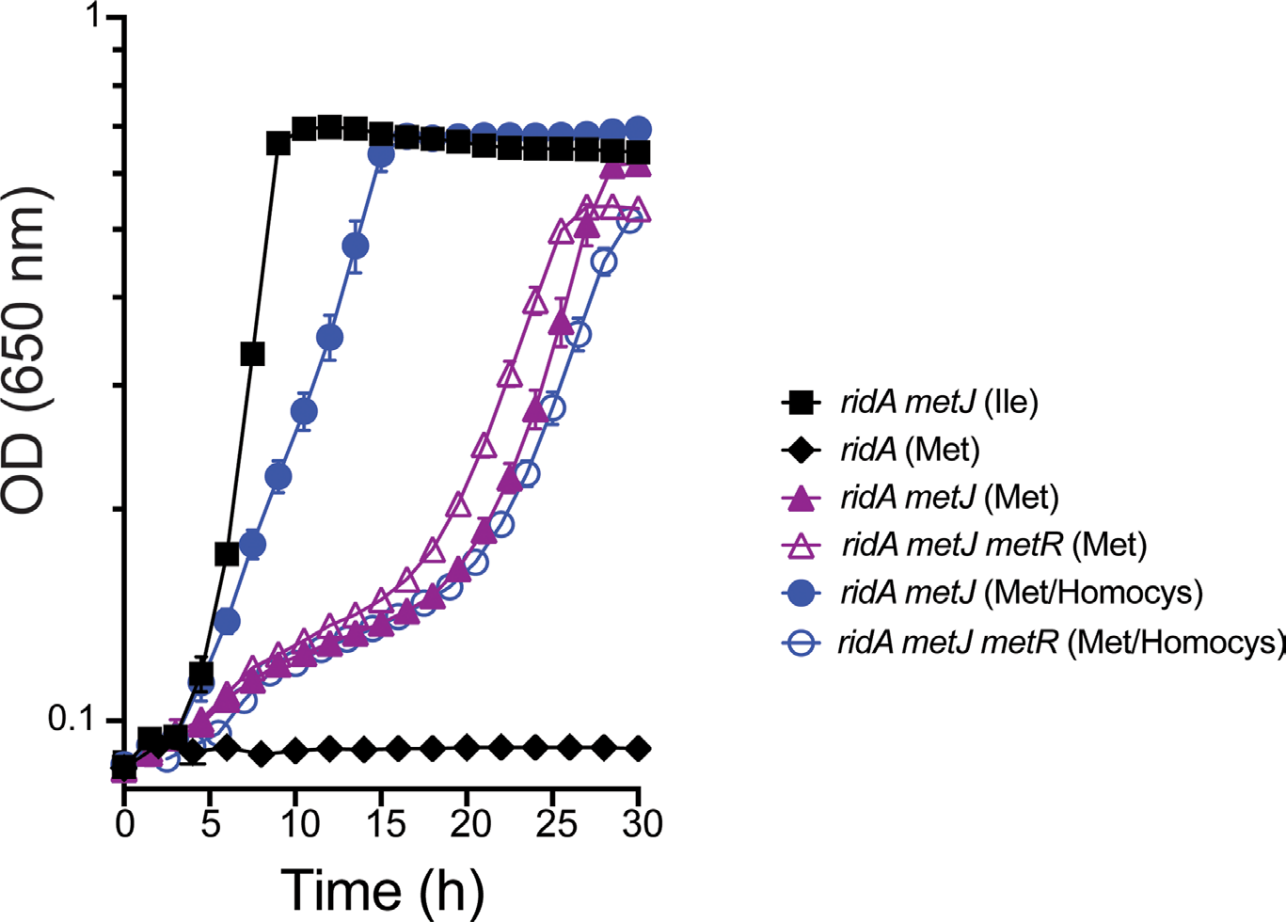
Stimulation of growth by homocysteine is MetR-dependent. All strains had an *alr* mutation and carried additional lesions as indicated. *ridA* (diamonds; DM17781), *ridA metJ* (solid squares/circles/triangles; DM17984) and *ridA metJ metR* (open circles/triangles; DM18542) were grown on minimal glucose medium with serine (5 mM) and additional supplements as indicated: Met (methionine, 0.3 mM) and Homocys (homocysteine, 0.2 mM). Data shown are averages from three biological replicates with standard error bars.

In addition to methionine biosynthetic genes, MetJ represses the transcriptional dual regulator MetR. Since increased methionine production did not explain the effect of a *metJ* deletion on the growth of the *ridA* mutant with exogenous serine (Fig. 3), we explored whether the suppression was MetR-dependent. A *metR* deletion was constructed and transduced into the relevant strains, and growth was monitored on minimal glucose medium supplemented with serine and other nutrients (Fig. 4). Several points were taken from these analyses. A *metR* mutation results in a methionine requirement due to decreased expression of *metE* in the absence of MetR [36]. Addition of methionine did not impact the growth of an *alr ridA metJ* mutant, and a *metR* deletion had no effect. These data showed that there was a level of growth suppression by a *metJ* mutation that was not MetR-dependent. The growth allowed by a *metJ* mutation was intermediate between that allowed by isoleucine (i.e. eliminated 2AA stress) and no addition. Homocysteine is the immediate precursor to methionine and also modulates the DNA binding of MetR [37]. When homocysteine was added to the medium, growth of the *alr ridA metJ* mutant was enhanced to the level allowed by isoleucine or a WT *ridA* locus. This growth stimulation occurred with or without supplementation of methionine, but only in the absence of *metJ* (Fig. 4, data not shown). Dramatically, a *metR* mutation eliminated growth stimulation by homocysteine. In total, these data showed that a *metJ* deletion suppressed the growth defect of an *alr ridA* mutant on exogenous serine by two mechanisms. One was dependent on homocysteine and MetR, and one was independent of both.

## Discussion

The RidA paradigm of 2AA stress has been a valuable model to explore how metabolic networks can respond to perturbation. *S. enterica* strains lacking *ridA* grow poorly on minimal media when (i) l-alanine is provided as the sole nitrogen source, (ii) pyruvate is provided as the sole carbon source or (iii) l-serine is added. Genetic analyses have helped define the RidA paradigm, with most spontaneous suppressor mutations reducing 2AA generation and/or accumulation [5,13,16]. Herein, we found that eliminating the transcriptional regulator MetJ suppressed three growth defects caused by 2AA-dependent phenotypes of a *ridA* mutant. A lesion in *metJ* derepresses methionine biosynthetic genes [23,24, 30], suggesting that increased methionine could be a part of the suppression mechanism. A *metJ* mutation did not reduce 2AA accumulation in a *ridA* mutant, meaning the suppression mechanism(s) involved remodelling of the metabolic network to allow growth in the relevant conditions. While lack of growth in each condition was 2AA-dependent, the mechanism of suppression by a *metJ* mutation had features unique to each.

### Suppression of *ridA* growth defect with pyruvate as carbon source

The inability of a *ridA* mutant to grow with pyruvate as the sole carbon source is due to the 2AA generated from endogenous serine [27]. A *metJ* mutation increased the growth of a *ridA* mutant to about half the level allowed by addition of isoleucine, which acts to eliminate 2AA. This level of suppression could also be attained by adding methionine to a *ridA* mutant, consistent with the *metJ* mutation acting by derepressing methionine synthesis. *ridA* mutants are limited for one-carbon units because of 2AA-mediated damage to GlyA [10]. Nutritional analyses supported a model in which the increase in methionine was needed to increase SAM biosynthesis, which in turn restored the synthesis of both the THZ and HMP moieties of thiamine. Each of these pathways includes a SAM radical protein, and their limitation has been characterized as a hallmark of SAM and/or iron sulphur cluster defects [31,32]. Importantly, neither the addition of methionine nor the lesion in *metJ* restored growth to the level that is achieved with the addition of isoleucine. This result is consistent with the finding of a previous study that, when grown with pyruvate, a *ridA* mutant displays a requirement for isoleucine. Excess pyruvate biases the branched-chain biosynthetic pathway to valine and leucine, and the 2AA-dependent inhibition of IlvE further constrains isoleucine synthesis [27]. Taken together, the data indicate that the lack of growth of *ridA* mutants on pyruvate is a composite of decreased activity of both IlvE and GlyA.

### Suppression of the serine sensitivity of *ridA* mutants

The addition of serine generates a more extreme 2AA burden than the other two conditions where endogenous serine pools are the source of the stressor. Methionine did not restore the growth of a *ridA* mutant in the presence of added serine, which distinguished the suppression mechanism from the one on pyruvate. A *metJ* mutation restored ~50% growth to a *ridA* mutant when exogenous serine was present. Without a stimulatory effect by methionine, there was not a simple scenario to explain the restored growth. Suppression by a *metJ* mutation on minimal with serine was not MetR-dependent, indicating that the increased level of MetR in a *metJ* mutant did not contribute to this suppression. In contrast, the increased growth of the *metJ ridA* mutant allowed by homocysteine was MetR-dependent. Thus, a *metJ* mutation increased growth in the presence of serine (i.e. 2AA stress) by two mechanisms: 50% growth stimulation was MetR-independent, while the full growth allowed by the *metJ* mutation and homocysteine was MetR-dependent. While these data suggested that increased regulatory activity of MetR was responsible for the growth stimulation, it remains unclear what component of the MetR regulon is involved in this effect. The MetR regulon includes *glyA* [38], but overexpression of *glyA* in trans failed to produce the effect of homocysteine, which seemed to eliminate a simple scenario.

### Growth with L-alanine as the sole nitrogen source

Because a *metJ* mutation does not reduce 2AA, the damage incurred by DadX limits the growth that can be achieved by remodelling the metabolic network. The data are consistent with the lack of growth on l-alanine being comprised primarily of the 2AA damage to DadX and to GlyA. In this scenario, the *metJ* mutation partially overcomes the compromised GlyA activity to allow the growth stimulation detected. Consistently, the growth of a *ridA* mutant on l-alanine was somewhat improved in the presence of exogenous glycine (OD_650_ ~0.3 after 35 h of continuous growth, compared to <0.1 without glycine addition).

In total, our experiments implicated at least three distinct mechanisms by which a *metJ* deletion could improve the growth of a *ridA* mutant: (i) de-repression of methionine biosynthesis, (ii) a MetR-independent mechanism that could not simply be explained by increased methionine availability and (iii) a MetR-dependent mechanism that was bolstered by increased homocysteine availability. Importantly, the analyses described herein assumed that the measurement of 2AA via IlvE activity on minimal glucose served as an accurate reflection of 2AA levels in the relevant strains, and this did not change by growth condition. Measuring 2AA levels via IlvE activity in conditions of significant 2AA stress, including those described here, is not always possible due to the associated growth defects. We cannot fully eliminate the possibility that 2AA levels are decreased in some of the specific situations where growth was being measured. The intracellular concentration of metabolites (e.g. methionine and SAM) was not directly quantified herein, which may limit the strength of the conclusions proposed. Minor changes in metabolite concentration or flux can modulate metabolism to produce quantifiable phenotypes that provide insights into connections that exist in the metabolic network. An integrated biochemical genetic approach with both *in vivo* and *in vitro* components remains a viable way to new metabolic knowledge. Analyses of the RidA paradigm highlight the integration of diverse metabolic nodes, with single targets of 2AA stress (such as GlyA) generating broad ripple effects on downstream pathways (i.e. methionine and thiamine biosynthesis). Better defining the consequences of metabolic perturbations imposed upon the metabolic network, in the context of 2AA stress, can generate insights into the metabolic plasticity and biochemical integration inherent in the metabolic networks of bacteria.

## Abbreviations

2AA: 2-aminoacrylate
HMP: 4-amino-5-(hydroxymethyl)-2-methylpyrimidine
2KMV: 2-keto-3-methylvalerate
NB: Nutrient Broth
NCE: no-carbon E
OD_650_: OD at 650 nm
PLP: pyridoxal 5′-phosphate
RidA: Reactive intermediate deaminase A
SAM: S-adenosyl methionine
THZ: 4-methyl-5-(2-hydroxyethyl)-thiazole
